# Disruption of Foraging by a Dominant Invasive Species to Decrease Its Competitive Ability

**DOI:** 10.1371/journal.pone.0090173

**Published:** 2014-03-04

**Authors:** Fabian Ludwig Westermann, David Maxwell Suckling, Philip John Lester

**Affiliations:** 1 School of Biological Sciences, Victoria University of Wellington, Wellington, New Zealand; 2 The New Zealand Institute for Plant & Food Research, Christchurch, New Zealand; INRA-UPMC, France

## Abstract

Invasive species are a major threat to biodiversity when dominant within their newly established habitat. The globally distributed Argentine ant *Linepithema humile* has been reported to break the trade-off between interference and exploitative competition, achieve high population densities, and overpower nests of many endemic ant species. We have used the sensitivity of the Argentine ant to the synthetic trail pheromone (Z)-9-hexadecanal to investigate species interactions for the first time. We predicted that disrupting Argentine ant trail following behaviour would reduce their competitive ability and create an opportunity for three other resident species to increase their foraging success. Argentine ant success in the control was reduced with increasing pheromone concentration, as predicted, but interactions varied among competing resident species. These behavioural variations provide an explanation for observed differences in foraging success of the competing resident species and how much each of these individual competitors can increase their foraging if the competitive ability of the dominant invader is decreased. The mechanism for the observed increase in resource acquisition of resident species appears to be a decrease in aggressive behaviour displayed by the Argentine ant, which may create an opportunity for other resident species to forage more successfully. Our demonstration of species interactions with trail pheromone disruption is the first known case of reduced dominance under a pheromone treatment in ants.

## Introduction

Invasive species are considered to be a major threat to biodiversity and can drive native species to extinction through competitive exclusion [1] or niche displacement [2], [3]. Invasive species can also have adverse effects on ecosystem functionality [4], and cause economic losses through decreased agricultural productivity and costs of control measures [4]–[6].

Several tramp ant species are among the 100 of the world's worst invasive species [7]. Invasive tramp ants can drastically change the structure and community dynamic of an ecosystem [8], [9]. They have also been reported many times to replace native ant species [10]–[12] without providing the same ecosystem services for plants and other community members [13], [14].

In ants, a well-defined ecological classification of three competition hierarchy levels has been established [15], [16], distinguishing among dominant, subdominant and subordinate species. Dominant species often place severe limitations on the foraging success of subordinate species at local food resources [17]–[23]. Behavioural traits [24] play an important role for the success of invasive species [25], [26] and may also be a factor for biotic resistance as the reactions of local community species influence the likelihood of native species to engage and interfere with the invader [27], [28].

Originally native to South America, the Argentine ant *Linepithema humile* has successfully become a global invader [9], [29]–[31]. It quickly monopolizes all resources in the local habitat [9], [32] and causes large reductions in abundances of native ants. The mechanisms to explain the Argentine ants' ability to displace native ant has been attributed to their capability of breaking of the trade-off usually involved between interference and exploitative capacities [1], [33], thus attaining ecological dominance [10], [34]–[36]. However there have also been reports of biotic resistance of native species against Argentine ants [1], [27], which use physical aggression or chemicals to successfully defend themselves and their territory.

Like many social insects, Argentine ants utilize a trail pheromone-based communication system to mark routes to valuable resources and exploit them efficiently. This trail-pheromones signal was demonstrated to deteriorate within approximately 30 minutes [37]. It has been hypothesized that one of the causes for the Argentine ants' success and their high impact on native species is their effective recruitment system [9]. This recruitment allows them to quickly divert workers from almost-depleted to newly discovered and presumably more worthwhile resources, thus gathering more food and enabling/supporting greater population abundance and thereby exploitatively outcompeting other resident species.

Previous laboratory and field tests have demonstrated that the synthetic trail pheromone (Z)-9-hexadecenal (Z9-16:Ald) disrupts Argentine ant trail-following behaviour by interfering with track angles to reduce foraging success [38]–[41]. High pheromone concentrations prevent Argentine ants from establishing stable trails and recruiting to resources in the area [39], although existing heavy traffic requires more pheromone for disruption [40]. Study designs to measure the impacts of invaders usually focus on before/after and control/impact comparisons, supported by experimental manipulations to maintain or decrease invasive populations and observe the effects on the local ecosystem and communities [42]–[45]. However, the Argentine ants and their sensitivity to synthetic trail pheromone provide an opportunity for a different approach to measure the impacts of an invasive species by altering their competitive abilities, rather than excluding them or comparing invaded with non-invaded areas.

Here, we attempt to reduce the competitive ability of the dominant Argentine ant using pheromones and assess the impact of these pheromones on the foraging success of competing community species. Firstly, we hypothesized that with increasing pheromone concentrations, Argentine ants would experience reduced resource acquisition, thus creating an opportunity for other resident species to increase their foraging success. Secondly, we hypothesized that increasing concentrations of synthetic pheromone would affect the foraging of co-occurring species differently, depending on the behavioural reactions between Argentine ants and the co-occurring species. Finally, as a mechanism for the increased foraging success of resident species in the presence of synthetic trail pheromone, we hypothesized that increasing pheromone concentrations could influence Argentine ant worker interactions with other species, with a variation in behavioural response between species.

## Methods

### Location

Experiments were conducted in an urban district of Lower Hutt, on the southern North Island of New Zealand, where Argentine ants have been well established since 2001. Three sites in the invasion zone (−41.222, 174.872; −41.221, 174.872 and −41.219, 174.879) were selected for competition experiments. No specific permissions were required to access the first two sampling sites, as they were on public ground. The last site was the garden of a private property, the owners gave oral permission to use their garden. None of the experiments involved any endangered or protected species. A preliminary survey had detected the highest abundance of individual workers for Argentine ants and the presence of other resident species at these locations. The three ant species encountered to approach food items together with Argentine ants were the native *Monomorium antarcticum*, the introduced *Ochetellus glaber* and the introduced *Technomyrmex jocosus*, with relative species composition varying between the three sites. None of the species are endangered or protected. Experiments were conducted on dry and sunny days between November 2011 and April 2012.

### Resource Competition Experiments

To assess the foraging success of *L. humile* under the influence of the pheromone treatment, 50 small marzipan pieces (average weight 0.024 g±0.012 g) were scattered on paper bait cards with 10 cm radius and placed haphazardly in an area of 10×10 m at the three sites. Marzipan (Odense Marzipan, Andre Prost, Inc.) was selected as the food because its compounds (∼30% sugar, glycose syrup, almonds/almond oil) were found to be highly attractive for Argentine and a range of other ants in previous trials. Only one bait card was made available for foraging per plot at any point during the experiment. A fresh bait card was placed at a new location 5 minutes after the last experiment had concluded and was placed at least 3 meters away from the previous location to prevent habituation. Bait cards were treated with four droplets (25 µl each) of ethanol solutions of synthetic trail pheromone at concentrations of 100 µg/µl, 1 µg/µl, 0.01 µg/µl and a control with pure solvent. Six replicates were carried out in random order for each of the three treatments and the control on each of the three sites. One droplet was made on each side of the bait card to provide an equal distribution of the pheromone. Bait cards were observed until all 50 food items had been picked up and carried over the edge of the bait card by ants (average time 85.9 min±2.3 min). For each individual food item that was being carried away by an ant worker from the bait card, the species of the worker carrying it was noted. Workers of all ant species were observed to be physically able to carry food items away. Due to differences in species abundance only one other resident species was primarily competing with Argentine ants at each location.

Logistic regression was used to compare the number of food items taken by each species with pheromone concentrations. The dependent response variable was whether an individual food item had been taken or not by Argentine ants at the end of each trial using pheromone concentration and competing ant species as explanatory variables. A binary logistic regression fits the data to give predicted probabilities (and odds) of whether a food item is taken, given particular values of the explanatory variables. For details see [46].

### Behavioural Scoring

To assess the difference in how encountered species would be treated by the Argentine ant and assess differences in their reaction in return, Argentine ant and workers of competing species were scored individually for both participating ants during encounters. At times between 1 to 5 minutes, a worker ant of *M. antarcticum*, *O. glaber* or *T. jocosus* entering the bait card was randomly chosen and its behaviour during its next interaction scored, as well as the behaviour displayed by the Argentine ant worker it was interacting with. Behaviour scores were taken: 0 = no interest or aggression; 1 = interest shown via antennation; 2 = ant retreats quickly; 3 = lunging, biting or leg-pulling, rising of gaster and exuding venom; 4 = prolonged (>5 s) incidences of aggression, individuals locked together and fighting [10]. Ants were considered “meeting” each other when they either touched each other physically, or when they were in close proximity (0.5 cm) to each other and both parties could be observed to display behaviours which could reasonably assumed to be addressed towards the other worker ant (for example stopping, antennating towards the other ant, turning around and quickly retreating). For half of the bait cards for each treatment and location, interactions of at least 10 individual ant workers were scored and no behaviour scores were taken after all food items had been removed by ants.

To investigate if Argentine ant behaviour in interactions would be influenced by the synthetic pheromone, the average of the behavioural scores was analysed with a Kruskal-Wallis test. Logistic regression was then used to test how interactions differ depending on species. The dependent response variable was whether an interaction was non-aggressive (scores 0, 1 and 2) versus aggressive behavioural (scores 3 and 4) with pheromone concentration and competing ant species as explanatory variables. Reactions of Argentine ant workers towards the competing species (*M. antarcticum*, *O. glaber* and *T. jocosus*) were tested as well as scores of the four species towards Argentine ant workers respectively. All data analysis was conducted using PASW Statistics v. 18.0. The data package associated with this manuscript has been made available at the Dryad data depository (Dryad accession number: doi: 10.5061/dryad.m64hs).

## Results

### Resource Competition Experiments

Our first hypothesis was that Argentine ants would experience reduced resource acquisition with increasing pheromone concentration, which disrupts their foraging. Argentine ant workers were observed to typically arrive first at the bait cards in all replicates, concurrent with the high exploitative abilities of these ants reported in previous publications [1], [10], [33]. The first scouts usually arrived within 2 to 18 minutes of the trial and substantially increased in number after that over the next 15 minutes. Although we did not quantify trail following behaviour or trail integrity, our observations indicate that Argentine ants always formed distinctive trails on and around the bait cards in controls, which were less distinctive in pheromone treatments of 1 µg/µl or higher. Furthermore in our observations during the experiment, Argentine ant workers appeared to take a slower approach on the bait card when pheromone was applied, often standing at the edge of the card for minutes and cleaning their antennae or walking in small circles before discovering food items. Workers of the three other species arrived much later, with the first worker ant picking up a food item between 14 to 31 minutes after the beginning of the experiment. By that time Argentine ants were nearly always already present in superior numbers and had started to retrieve food items, but this was reduced with increasing pheromone concentration.

**Figure 1:**
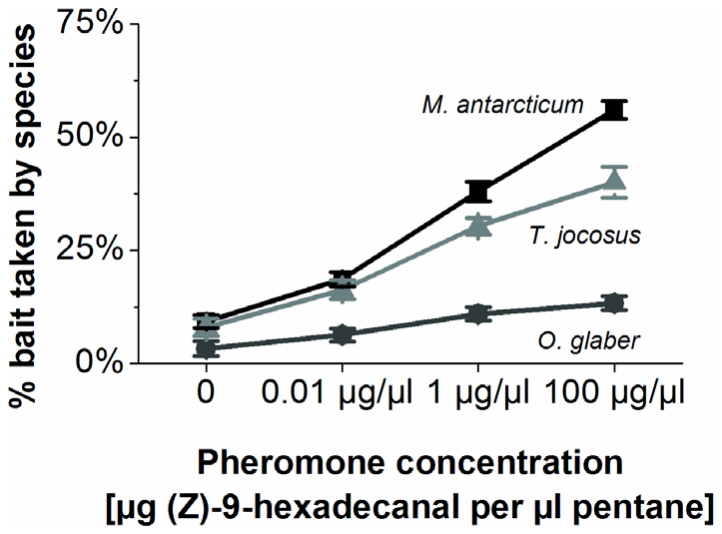
Food items taken by ants. Percentage of average number of food items taken in trials (n = 6 per treatment and site) by *M. antarcticum*, *O. glaber* and *T. jocosus* from Argentine ants depending on the treatment with different concentrations of synthetic trail pheromone (Z)-9-hexadecanal (measured in µg pheromone per µl solvent). Error bars represent standard error of average food items taken.

Logistic regression indicated that the trail pheromone had a substantial impact on the competition between Argentine ants and the three tested species *M. antarcticum*, *O. glaber* and *T. jocosus*. The synthetic trail pheromone affected the Argentine ants' ability to dominate the bait cards and retrieve food items, but the effect was considerably stronger at higher pheromone concentrations. The number of food items retrieved by each of the three species was significantly higher with each increase in pheromone concentration (control vs. any of the pheromone treatments *P*<0.001). There was also a significant difference for the number of food items taken between species for each concentration level (Figure 1, Table 1). The native ant *M. antarcticum* appeared to benefit the most from the pheromone treatment and increased the number of collected food items from an average of 4.6 in the controls to 28 at a treatment of 100 µg/µl (a 608% increase). The exotic ants *T. jocosus* increased their foraging success from an average of 4 to 20 (a 500% increase) and *O. glaber* increased from an average of 1.6 retrieved food items to 6.7 (a 416% increase).

**Table 1:** Statistics for number of food items taken.

	ß	S.E.	Wald	df	*P*	Exp(ß)
*O. glaber* vs. *M. antarcticum*	0.39	0.10	15.77	1	0.001 **	1.47
*T. jocosus* vs. *M. antarcticum*	1.68	0.13	178.27	1	0.001 **	5.35
0.01 µg/µl vs. Control	−0.79	0.17	22.91	1	0.001 **	0.45
1 µg/µl vs. Control	−1.65	0.15	113.94	1	0.001 **	0.19
100 µg/µl vs. Control	−2.16	0.15	201.34	1	0.001 **	0.12
Constant	2.11	0.14	226.75	1	0.001 **	8.23

* *P*<0.05;

** *P*<0.01.

Logistic regression on the number of food items taken in trials by Argentine ants versus *M. antarcticum*, *O. glaber* and *T. jocosus*. First two rows show the difference between food items taken by Argentine ants from a species, with *M. antarcticum* as reference. The next three rows show the difference of food items taken by Argentine ants depending on pheromone concentration with the control treatment as a reference. ß is the log-odds units, S.E. the standard errors associated with the coefficients, Wald the Wald chi-square value, df the degrees of freedom, *P* the statistical significance and Exp(ß) the odds ratio.

### Behavioural Scoring

**Figure 2:**
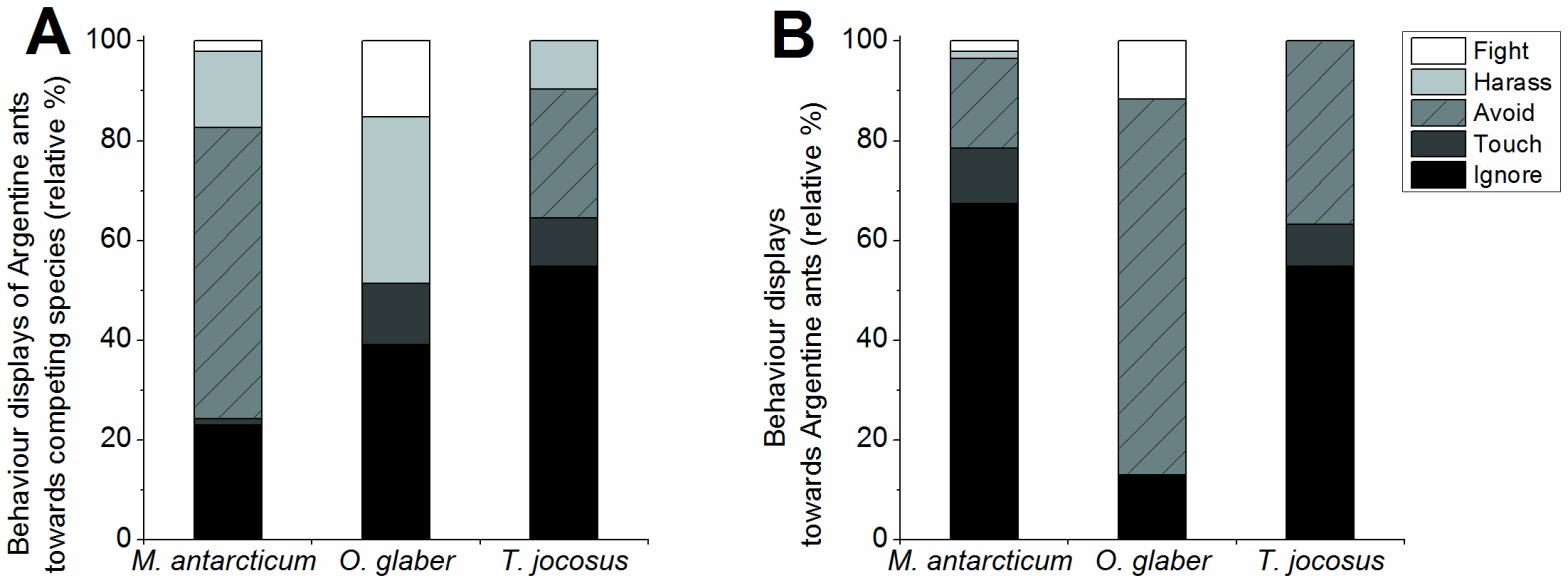
Relative percentage of behavioural scores during interactions. (A) Argentine ants towards *M. antarcticum*, *O. glaber* and *T. jocosus* and (B) *M. antarcticum*, *O. glaber* and *T. jocosus* towards Argentine ants. Numbers surrounding each graph (0–4) represent the displayed behaviours during interactions, which was scored as 0 = no interest or aggression; 1 = interest shown via antennation; 2 = ant retreats quickly; 3 = lunging, biting or leg-pulling, rising of gaster and exuding venom; 4 = prolonged (>5 s) incidences of aggression, individuals locked together and fighting. Small numbers and dashed lines mark intervals of 0, 25, 50, 75 and 100 for the percentage of interactions during which these behaviours were being displayed.

We had hypothesized that behavioural interactions between Argentine ants and the co-occurring species would be different for each species, affecting foraging of co-occurring species differently. The average behavioural score for Argentine ant behaviour towards their interaction partner was significantly different between the three species (Kruskal-Wallis *P*<0.01), as was the reaction of *M. antarcticum*, *O. glaber* and *T. jocosus* in return (Kruskal-Wallis *P*<0.01) (Figure 2). Workers of the native ant *M. antarcticum* were often avoided (aggression score 2) by Argentine ants, which retreated from *M. antarcticum* workers even when outnumbering it (Figure 2A). In return, *M. antarcticum* workers were often observed to ignore Argentine ants (aggression score 0), often walking on the bait card without any sign of hesitation, picking up a food item and leaving again even when harassed (biting, leg pulling) by Argentine ant workers on rare occasions (Figure 2B).

Workers of the smaller exotic ant *O. glaber* were treated with considerably higher aggression by Argentine ants when encountered. The Argentine ants actively engaged and chased the smaller ants off the bait card. No occurrence of avoidance behaviour (aggression score 2) by Argentine ant workers towards *O. glaber* workers was ever observed (Table 2). In return the *O. Glaber* workers never displayed any touching (aggression score 1) or harassment (aggression score 3) (Table 3) and appeared to show great hesitation to go near the food items when they appeared to perceive Argentine ant workers in the vicinity, often stopping and turning immediately when coming as close as 0.5 cm to an Argentine ant worker.

**Table 2:** Total number of behavioural observations.

Species	Treatment	Argentine ants		Competitors	
		Non Aggressive	Aggressive	Non Aggressive	Aggressive
*M. antarcticum*	0	20	10	26	4
	0.01 µg/µl	28	5	33	0
	1 µg/µl	36	5	40	1
	100 µg/µl	35	5	40	0
*O. glaber*	0	11	18	23	6
	0.01 µg/µl	14	22	32	4
	1 µg/µl	23	15	34	4
	100 µg/µl	23	12	33	2
*T. jocosus*	0	25	6	31	-
	0.01 µg/µl	35	3	38	-
	1 µg/µl	35	3	38	-
	100 µg/µl	35	2	37	-

Total number of observations of non-aggressive (behaviour scores 0, 1 and 2) and aggressive (behaviour scores 3 and 4) behaviour displayed by Argentine ants towards each competing species and observations for this species in return, as a function of pheromone concentration or no treatment.

**Table 3:** Statistics for aggression by Argentine ants.

	ß	S.E.	Wald	df	*P*	Exp(ß)
*O. glaber* vs. *M. antarcticum*	1.558	0.287	29.485	1	0.001 **	4.751
*T. jocosus* vs. *M. antarcticum*	−0.718	0.363	3.918	1	0.048 *	0.488
0.01 µg/µl vs. Control	−.0566	0.340	2.771	1	0.096	0.568
1 µg/µl vs. Control	−1.108	0.352	9.921	1	0.002 **	0.330
100 µg/µl vs. Control	−1.287	0.366	12.398	1	0.001 **	0.276
Constant	−0.836	0.297	7.933	1	0.005 **	0.433

* *P*<0.05;

** *P*<0.01.

Logistic regression on displayed aggression by Argentine ants towards *M. antarcticum*, *O. glaber* and *T. jocosus*. The first two rows show the difference between aggressions of Argentine ants towards a species, with M. antarcticum as reference. The next three rows show aggression of Argentine ants depending on pheromone concentration with the control treatment as a reference. ß is the log-odds units, S.E. the standard errors associated with the coefficients, Wald the Wald chi-square value, df the degrees of freedom, P the statistical significance and Exp(ß) the odds ratio.

Workers of *T. jocosus* and the Argentine ant most frequently ignored and avoided each other, and while Argentine ant worker occasionally harassed *T. jocosus*, no occurrences of prolonged combat (aggression score 4) were observed between these species throughout the experiment. Additionally no instances of aggression from *T. jocosus* towards Argentine ants (behavioural score 3 or 4) were observed.

The results of the logistic regression (Table 3) analysing differences in non-aggressive versus aggressive behavioural reactions show significant differences between the aggression displayed by Argentine ants towards *M. antarcticum* and *O. glaber* (Wald = 29.485; df = 1; *P*<0.001), with *O. glaber* having a 4.75 times higher chance of being attacked than *M. antarcticum*. Between *M. antarcticum* and *T. jocosus*, the latter had an average of 0.49 chance of being attacked (Wald = 3.918; df = 1; *P* = 0.048). There was also a significant difference in the reactions of these species towards Argentine ants. Displayed aggression differed significantly between *M. antarcticum* and *O. glaber* towards Argentine ants (Wald = 6.148; df = 1; *P* = 0.013). The difference in aggression between *M. antarcticum* and *T. jocosus* towards Argentine ants could not be tested for with logistic regression, due to the absence of any aggression displayed by *T. jocosus* (no instances of scores 3 or 4 observed; (Table 2).

**Figure 3:**
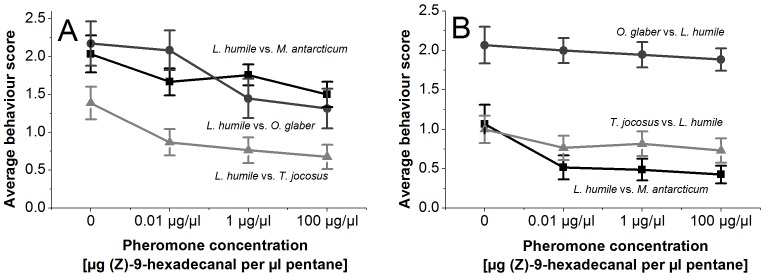
Average aggression scores of ants. Average aggression of (A) Argentine ants towards *M. antarcticum*, *O. glaber* and *T. jocosus* and (B) *M. antarcticum*, *O. glaber* and *T. jocosus* towards Argentine ants depending on the treatment with different concentrations of synthetic trail pheromone (Z)-9-hexadecanal (measured in µg pheromone per µl solvent). Aggression was scored as 0 = no interest or aggression; 1 = interest shown via antennation; 2 = ant retreats quickly; 3 = lunging, biting or leg-pulling, rising of gaster and exuding venom; 4 = prolonged (>5 s) incidences of aggression, individuals locked together and fighting. Error bars represent Standard Error of average aggression scores.

In our third hypothesis, we investigated if increasing pheromone concentrations could influence Argentine ant worker interactions with other species. The treatment with synthetic trail pheromone had a substantial impact on the behaviour displayed by Argentines ants towards *M. antarcticum*, *O. glaber* and *T. jocosus* (Figure 3A), lowering the average behavioural score (Kruskal-Wallis *P*<0.01). The behaviour displayed by *O. glaber*, *T. jocosus* and *M. antarcticum* appeared to be only slightly influenced by the application of pheromone, although all showed a small reduction (Figure 3B). The results of the logistic regression (Table 3) analysing differences in non-aggressive versus aggressive behavioural reactions reveal a highly significant reduction of displayed aggression by Argentine ants with pheromone concentrations of 1 µg/µl (Wald = 9.921; df = 1; *P*<0.001) and 100 µg/µl (Wald = 12.398; df = 1; *P*<0.001) compared to untreated controls. Furthermore, aggressive reactions of *M. antarcticum*, *O. glaber* and *T. jocosus* towards Argentine ants decreased with increasing pheromone treatments (Table 4).

**Table 4:** Statistics for aggression by *M. antarcticum*, *O. glaber* and *T. jocosus* towards Argentine ants.

	ß	S.E.	Wald	df	*P*	Exp(ß)
*O. glaber* vs. *M. antarcticum*	1.33	0.54	6.148	1	0.013 *	3.78
*T. jocosus* vs. *M. antarcticum*	−17.85	3251.82	0.000	1	0.996	0.000
0.01 µg/µl vs. Control	−1.278	0.632	4.090	1	0.043 *	0.279
1 µg/µl vs. Control	−1.13	0.59	3.712	1	0.054	0.32
100 µg/µl vs. Control	−2.032	0.804	6.385	1	0.012 *	0.131
Constant	−2.388	0.520	21.062	1	0.000 **	0.092

* *P*<0.05;

** *P*<0.01.

Logistic regression on displayed aggression by *M. antarcticum*, *O. glaber* and *T. jocosus* towards Argentine ants. The first two rows show the difference between aggressions by a species towards Argentine ants, with M. antarcticum as reference. The next three rows show aggression depending on pheromone concentration with the control treatment as a reference. ß is the log-odds units, S.E. the standard errors associated with the coefficients, Wald the Wald chi-square value, df the degrees of freedom, P the statistical significance and Exp(ß) the odds ratio.

## Discussion

Our data support the hypothesis that confusing a dominant invasive species and reducing its competitive ability can reduce its resource acquisition and create an opportunity for other resident species to increase their foraging. While dominating untreated bait cards, Argentine ants arrived sporadically and in a more disorganized manner, when treated with synthetic pheromone. This is consistent with results in previous studies [38]–[41], which had reported that trail following behaviour of Argentine ants could be disrupted with synthetic pheromone. These studies provided a proof of concept for the pheromone disruption in Argentine ants by analysing trail integrity [38] and providing evidence for a negative impact on their foraging [39], [41]. However, it was previously unclear whether this interference with Argentine ants recruitment actually translates into an effect on the resident ant community, which could be used to support native species. In our experimental approach the trail disruption gave co-occurring species an opportunity to locate and remove baits much more successfully than without the pheromone.

Argentine ants reacted differently to each of the three species, showing a variety of patterns in frequency and type of non-aggressive and aggressive behaviours towards their competitors. Similar observations have been made in other ant species, which react differently towards certain competitors [47], [48]. All three tested competing species *M. antarcticum*, *O. glaber* and *T. jocosus* were able to utilize the advantage arising through the previously reported confusion caused by the pheromone application and significantly increase their foraging success. However, the increase in the number of retrieved food items varied among species, with the relative increase of *M. antarcticum* being the most substantial. Comparatively with increasing pheromone concentration, *O. glaber* showed less dramatic increase.

The differences in foraging success can be well explained by the differences in behavioural traits [25] and their manifestations in interactions between Argentine ants and the three species. For example, *M. antarcticum* is frequently avoided by Argentine ants, which make way for approaching *M. antarcticum* workers even when outnumbering them. Workers of *M. antarcticum* mostly seemed to ignore Argentine ants and single *M. antarcticum* workers were frequently observed to walk on the bait card and retrieve a food item without hesitation, apparently uninterested in present Argentine ant workers. These behaviour patterns are well within expected interaction ranges, as *M. antarcticum* workers are larger than Argentine ant workers and, unless dramatically outnumbered [49], have been shown to annihilate Argentine ant workers in physical interactions by biting them in half. However, *M. antarcticum* are also slow moving and slower to recruit than Argentine ants, especially at higher temperatures [50], which may explain why Argentine ants are still dominant at food sources. While *O. glaber* does benefit from the pheromone disruption, their foraging success increases less than the other species, since their workers are frequently harassed or attacked by Argentine ant workers. Workers of *O. glaber* were frequently observed to retreat when near Argentine ant workers, even when Argentine ants were walking in circles, apparently confused, and not directly interacting with *O. glaber* individuals. Workers of *T. jocosus* and Argentine ants display a similar pattern of ignoring and avoidance towards each other, with some harassment being exhibited by Argentine ant workers. The observed behavioural patterns are concurrent with aggression and avoidance patterns appropriate to dominance ranks of these species within the ant community [15], [16], with the Argentine ant taking a dominant role, while *M. antarcticum* and *T. jocosus* are subdominant and *O. glaber* subordinate.

As a mechanism for increased foraging success of the three competing resident species, we observed decreased aggression being exhibited by dominant Argentine ant workers when treated with pheromone, combined with their reduced ability to locate the food items. Our data indicate a significant reduction in aggression displayed by Argentine ant workers with increasing pheromone concentrations. While previous studies have investigated changes of aggression in social insects related to cuticular hydrocarbons [51], [52], queen pheromones [53] and alarm pheromones [54], the impact on aggression due to experimental pheromone changes in a wider area are unexplored at this point. In the presence of synthetic pheromone, Argentine ants seemed to spread out more and no longer followed distinct trails, which could increase the chance of encounters between species. However, Argentine ant workers also more frequently stand around or move in small circles. This behaviour appeared to allow workers of the other species to pass them in close proximity (∼0.5 cm) without provoking any aggressive reaction and thus retrieving food more easily. We propose two non-mutually exclusive hypotheses to explain this reduction in aggressiveness. First the trail disruption caused by higher concentrations of trail pheromone could simply decrease the likelihood of Argentine ants workers being able to perceive the workers of other species, even at very close distance, thus ignoring them much more often. Alternatively high pheromone concentrations might alter the reactions of Argentine ants, making individual workers less aggressive. Ants frequently defend established trails to resources, so it might be possible that, due to the disruption of the trail, the territoriality of Argentine ants is reduced, which therefore decreases their aggressive behaviour within the area of the pheromones' influence.

We have demonstrated that it is possible to significantly reduce the foraging success and displayed aggression of the Argentine ant, a globally dominant invader [9], [29]–[31]. Argentine ants have been reported many times to competitively exclude subdominant and subordinate species due to their ability to quickly locate food and defend it efficiently [1], [10], [33]. Our experiments showed that it is possible to reduce the competitiveness of the Argentine ants by interfering with their ability to find and defend food sources. Species from a lower dominance hierarchy were able to take advantage of this opportunity. This provides evidence that pheromone-based control measures could help to stabilize or even increase local populations of native species that were previously being suppressed. Insecticides have often led to losses of non-target species [55], [56] and have recently shown to even have the potential of giving an advantage to invasive species [57]. Over a longer time, reduced foraging ability of Argentine ants could lead to reduced populations of this invader, potentially even to a point where the invaders' population collapses and they are replaced by other local species. Future experimental studies should focus on whether or not long-term reduction of competitive abilities of a dominant species is possible and results in anticipated changes in the species' community which could be utilized in invasive species management.

Our demonstration of species interactions with trail pheromone disruption is the first known case of reduced dominance from pheromone-based treatment in ants, and this area offers new prospects for behavioural interference in invasive species management.
